# Influence of soil moisture on codenitrification fluxes from a urea-affected pasture soil

**DOI:** 10.1038/s41598-017-02278-y

**Published:** 2017-05-19

**Authors:** Timothy J. Clough, Gary J. Lanigan, Cecile A. M. de Klein, Md. Sainur Samad, Sergio E. Morales, David Rex, Lars R. Bakken, Charlotte Johns, Leo M. Condron, Jim Grant, Karl G. Richards

**Affiliations:** 1 Department of Soil and Physical Sciences, Lincoln University, Lincoln, New Zealand; 2 Teagasc, Environmental Research Centre, Johnstown Castle, Wexford Ireland; 3 AgResearch Invermay, Mosgiel, New Zealand; 4 Department of Microbiology and Immunology, Otago School of Medical Sciences, University of Otago, Dunedin, New Zealand; 5 Department of Environmental Sciences, Norwegian University of Life Sciences, Ås, Norway; 6 Statistics and Applied Physics, Teagasc, Ashtown Dublin 15, Ireland

## Abstract

Intensively managed agricultural pastures contribute to N_2_O and N_2_ fluxes resulting in detrimental environmental outcomes and poor N use efficiency, respectively. Besides nitrification, nitrifier-denitrification and heterotrophic denitrification, alternative pathways such as codenitrification also contribute to emissions under ruminant urine-affected soil. However, information on codenitrification is sparse. The objectives of this experiment were to assess the effects of soil moisture and soil inorganic-N dynamics on the relative contributions of codenitrification and denitrification (heterotrophic denitrification) to the N_2_O and N_2_ fluxes under a simulated ruminant urine event. Repacked soil cores were treated with ^15^N enriched urea and maintained at near saturation (−1 kPa) or field capacity (−10 kPa). Soil inorganic-N, pH, dissolved organic carbon, N_2_O and N_2_ fluxes were measured over 63 days. Fluxes of N_2_, attributable to codenitrification, were at a maximum when soil nitrite (NO_2_^−^) concentrations were elevated. Cumulative codenitrification was higher (P = 0.043) at −1 kPa. However, the ratio of codenitrification to denitrification did not differ significantly with soil moisture, 25.5 ± 15.8 and 12.9 ± 4.8% (stdev) at −1 and −10 kPa, respectively. Elevated soil NO_2_^−^ concentrations are shown to contribute to codenitrification, particularly at −1 kPa.

## Introduction

The concentration of nitrous oxide (N_2_O) in the atmosphere has increased since 1750 due to human activity with values surpassing the highest concentrations recorded in ice cores during the past 800,000 years, and exceeding the pre-industrial level by 20%^1^. Reductions in the anthropogenic forcing of Earth’s climate system and the recovery of the ozone layer would be enhanced if anthropogenic emissions of N_2_O were reduced^1,2^. However, the atmospheric N_2_O concentration continues to increase, predominately due to agricultural intensification, with 80% of the increase resulting from increased fertilizer use and manure applications for the purpose of food production^3^. Nitrous oxide emissions from grazed grasslands make a significant contribution to anthropogenic N_2_O emissions^4,5^ as a consequence of ruminant urine patches supplying nitrogen (N) substrate that is in excess of the pasture sward’s N requirement^6,7^. Emissions of N_2_O from pastures result from microbial transformations of N substrates applied via nitrification, nitrifier-denitrification, heterotrophic denitrification (hereafter referred to as denitrification unless otherwise stated), and codenitrification^8–10^. A further significant consequence of denitrifying mechanisms is the production and loss of dinitrogen (N_2_). Although environmentally benign, N_2_ losses lead to poor N use efficiency and reduced production, resulting in economic losses through the need to add further inorganic N. While reactive N (Nr) losses, such as nitrate (NO_3_^−^) leaching and ammonia (NH_3_) volatilization, are well researched, the loss of N_2_ from pasture systems is poorly studied and often only identified by default via the application of N balance methods^11^. For example, of the N applied to grasslands some 20–40% is typically unaccounted for and assumed to be lost as N_2_^11–13^. Therefore, methods to reduce emissions of both N_2_O and N_2_ require a better understanding of the emission pathways.

Shoun *et al*.^14^ and Tanimoto *et al*.^15^ first described codenitrification after demonstrating, with ^15^N tracer, that N_2_O and N_2_ production occurred in a different manner to the routinely accepted pathways of nitrification and denitrification. It has been suggested that codenitrification results from microbially mediated N-nitrosation reactions^14–16^. Codenitrification is one of the least studied N loss pathways and its contribution to agricultural N_2_O and N_2_ emissions remains unclear^17^.

**Figure 1:**
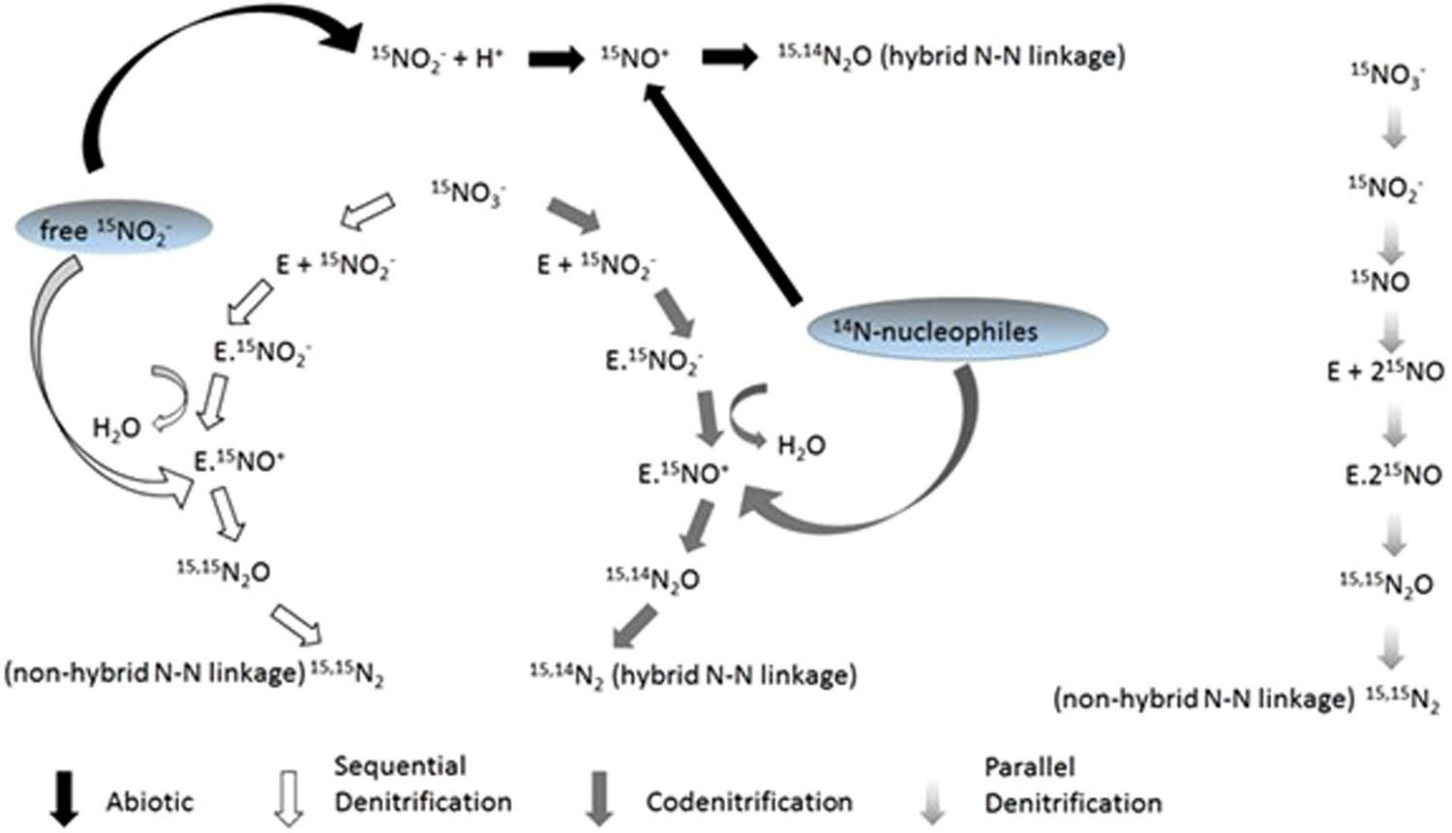
Simplified diagram (adapted from Spott *et al*.^16^, Weeg-Aerssens *et al*.^18^, Schmidt *et al*.^55^) showing abiotic denitrification, parallel denitrification, sequential denitrification and codenitrification pathways. During abiotic production an electrophile (e.g. the nitrosonium cation NO^+^ which is formed under acidic soil conditions) replaces the hydrogen atom of a nucleophile with a hybrid N-N bond formed following deprotonation. The parallel pathway results in a non-hybrid N-N bond as the result of two NO_2_^−^ or two NO molecules being bound, simultaneously to one enzyme (E), which theoretically excludes the possibility of a nitrosation reaction occurring and the formation of a hybrid N-N bond^55,56^. However, a two-step process occurs in the sequential pathway when NO_2_^−^ or NO molecules initially bind to an enzyme (E) followed by a free NO_2_^−^, or NO molecule, (originating from the original NO_3_^−^ pool) reacting with the enzyme complexed N species to form a non-hybrid N-N bond. The two-step sequence also permits the enzyme complexed N species to function as an electrophile which is able to be to be attacked by nucleophiles producing a hybrid N-N bond. Nucleophiles able to partake in codenitrification reactions include amines, ammonium, hydrazine, and ammonia.

Codenitrification is a process that co-metabolises organic N compounds, such as amines, to produce N_2_O and/or N_2_, and is also referred to as biotic N-nitrosation^16^. Codenitrification involves the replacement of a hydrogen atom in an organic compound with a nitroso group (—N=O). Under near neutral to alkaline soil pH conditions, common to pasture soils, codenitrification may occur via enzymatic catalysis (Fig. 1), with enzymatic nitrosyl compounds (E-NO^+^ or E-NO) attracting nucleophilic compounds^16,18^. Nucleophiles involved in codenitrification include hydroxylamine, ammonium (NH_4_^+^), hydrazine, amino compounds, and ammonia (NH_3_). The resulting gas products formed, N_2_O or N_2_, contain one N atom originating from the inorganic-N (e.g. NO_2_^−^), and a second atom from the co-metabolised organic compound^16,18^. Significant rates of both partial and complete codenitrification are only likely to occur if nucleophile concentrations are at least one or two orders of magnitude greater than that of NO_2_^−^ and NO^16^.

Heterotrophic denitrification results in the reduction of NO_3_^−^ to N_2_ with nitrite (NO_2_^−^), nitric oxide (NO), and N_2_O obligate intermediaries^19^ (Fig. 1). Formation of the N_2_O molecule is recognized as occurring via parallel or sequential pathways^16^ and references therein. In the parallel pathway simultaneous bonding of two NO_2_^−^ or two NO molecules to an enzyme, where both NO_2_^−^ and NO are derived from the same NO_3_^−^ source, creates a non-hybrid N-N bond, thus precluding the occurrence of codenitrification^16^. However, a two-step reaction, the sequential pathway, results in either NO_2_^−^ or NO initially bonding with an enzyme, which in turn may react with either free NO_2_^−^ or NO to form a non-hybrid N-N bond, or alternatively, this enzyme bound N can act as an electrophile and react with nucleophiles (e.g. amines) to form a hybrid N-N bond (Fig. 1). Consequently, hybrid N-N gas production, codenitrification, can occur simultaneously as a result of conventional denitrification (Fig. 1)^16^. Formation of hybrid N_2_ has also been reported to occur when NH_3_, hydrazine (N_2_H_4_) or amines are co-metabolised during codenitrification^20^.

Abiotic nitrosation is also a well-recognized phenomena^21,22^. In abiotic reactions, free NO_2_^−^ derived from nitrification or denitrification processes is chemically transformed to produce the nitrosonium cation (NO^+^) under acidic conditions. The NO^+^ cation reacts with a nucleophile (e.g. amine) to produce a hybrid N-N linkage (Fig. 1)^16^ and references therein. This process differs from codenitrification since the formation of the NO^+^ electrophile is chemically dependent on the soil pH and involves free NO_2_^−^ in the soil solution as the precursor. Nucleophiles involved in abiotic reactions include hydroxylamine, NH_4_^+^, hydrazine, amines, and NH_3_. However, relatively high soil pH values under grazed pasture conditions mean that the equilibrium concentrations of free nitrosating agents are generally inadequate for abiotic nitrosation to be significant^16^.

In grazed pastures ruminant urine deposition onto pasture soil temporarily elevates soil pH following urea hydrolysis, creating a urinary-N cascade that produces potential nucleophiles (e.g. NH_4_^+^ and NH_3_) at high concentrations. Simultaneously, enzyme bound nitrosating agents (E-NO^+^ or E-NO), may be formed during denitrification of nitrate (NO_3_^−^) or as supplied by NO_2_^−^ or NO during processes such as nitrification of nitrifier-denitrification^19^. Thus urine patches are potentially conducive to codenitrification occurring. In the only *in vivo* study to date to focus on codenitrification, Selbie *et al*.^23^ confirmed the occurrence of codenitrification within ruminant urine-affected pasture soil with 95% of the N_2_ emitted over 123 days resulting from codenitrification, with N_2_ the dominant product, and where the codenitrified N_2_ was equivalent to 56% of the N applied. This experiment by Selbie *et al*.^23^ received regular rainfall and it may be that the dominance of codenitrified N_2_ over codenitrified N_2_O may have been the result of, as the authors suggest, hybrid N_2_O being converted to hybrid N_2_ via heterotrophic denitrification (Fig. 1). Conceptually, the recognized environmental constraints on denitrification should also apply to codenitrification^16^, since codenitrification depends on enzyme bound nitrosyl compounds, formed during denitrification, being present (Fig. 1). A key driver of denitrification is the soil’s oxygen status, and wetter soils result in higher levels of anaerobiosis since oxygen diffuses 1 × 10^4^ times slower through water when compared to air^24^. Thus wetter soils should have higher rates of codenitrification. In order to test this hypothesis, and better understand the constraints and importance of codenitrification in pasture soils, we performed an experiment using either saturated soil or soil at field capacity to determine relative rates of codenitrification. The objective of the study was to investigate the effect of soil moisture on the rate of codenitrification from simulated urine applied to a free draining permanent grassland soil.

## Results

### Soil moisture, pH, DOC and inorganic-N

**Figure 2:**
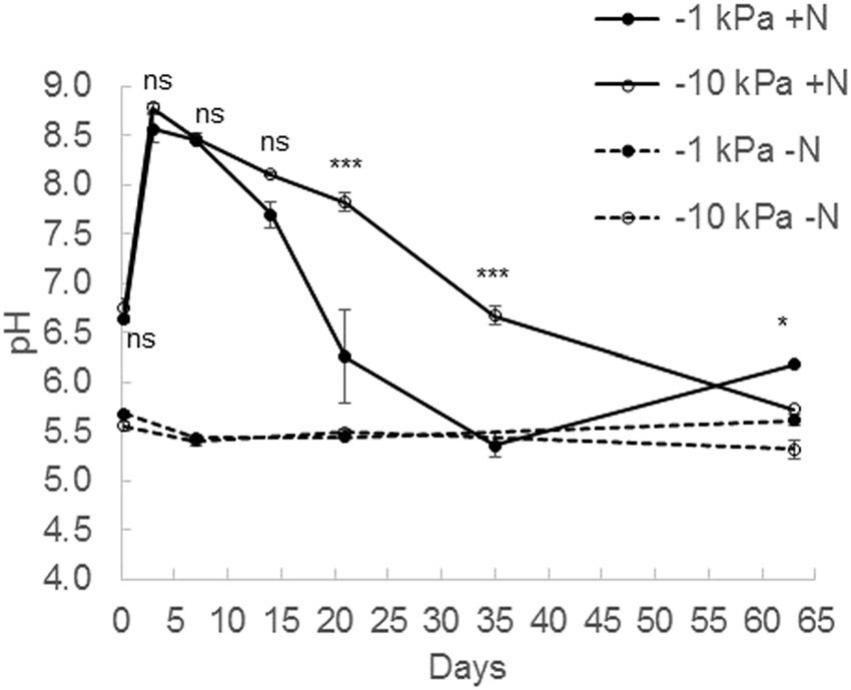
Changes in soil pH over time. Soil pH under near saturated (−1 kPa) or field capacity (−10 kPa) soil moisture conditions, following urea application (+N) or nil urea application (−N). Symbols are means (n = 4) with vertical error bars the standard error of the mean. Asterisks *^,^**^,^***indicate significant differences between moisture treatments under urea treatments at P < 0.05, P < 0.01, and P < 0.001, respectively.

**Figure 3:**
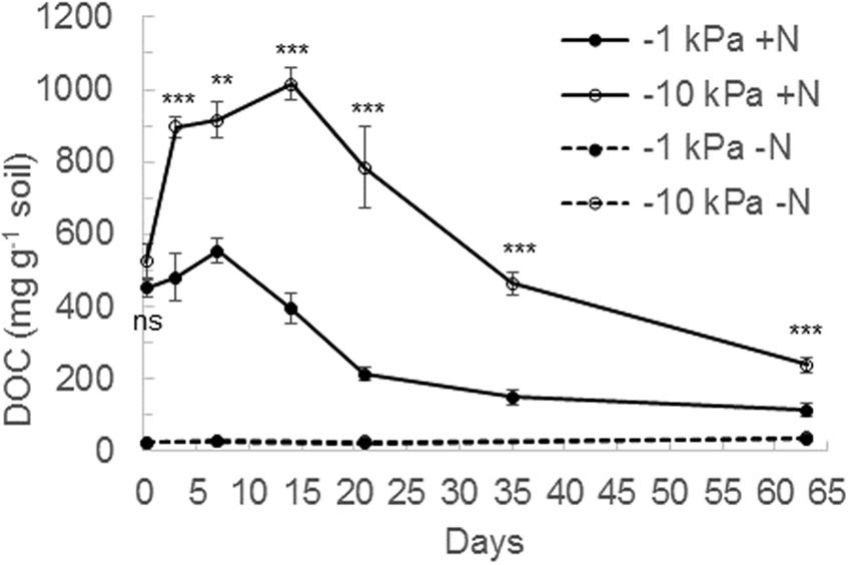
Changes in soil cold water extractable organic carbon (DOC) over time. Concentrations of soil DOC under near saturated (−1 kPa) or field capacity (−10 kPa) soil moisture conditions, following urea application (+N) or nil urea application (−N). Symbols are means (n = 4) with vertical error bars the standard error of the mean. Asterisks *^,^**^,^***indicate significant differences between moisture treatments under urea treatments at P < 0.05, P < 0.01, and P < 0.001, respectively.

**Figure 4:**
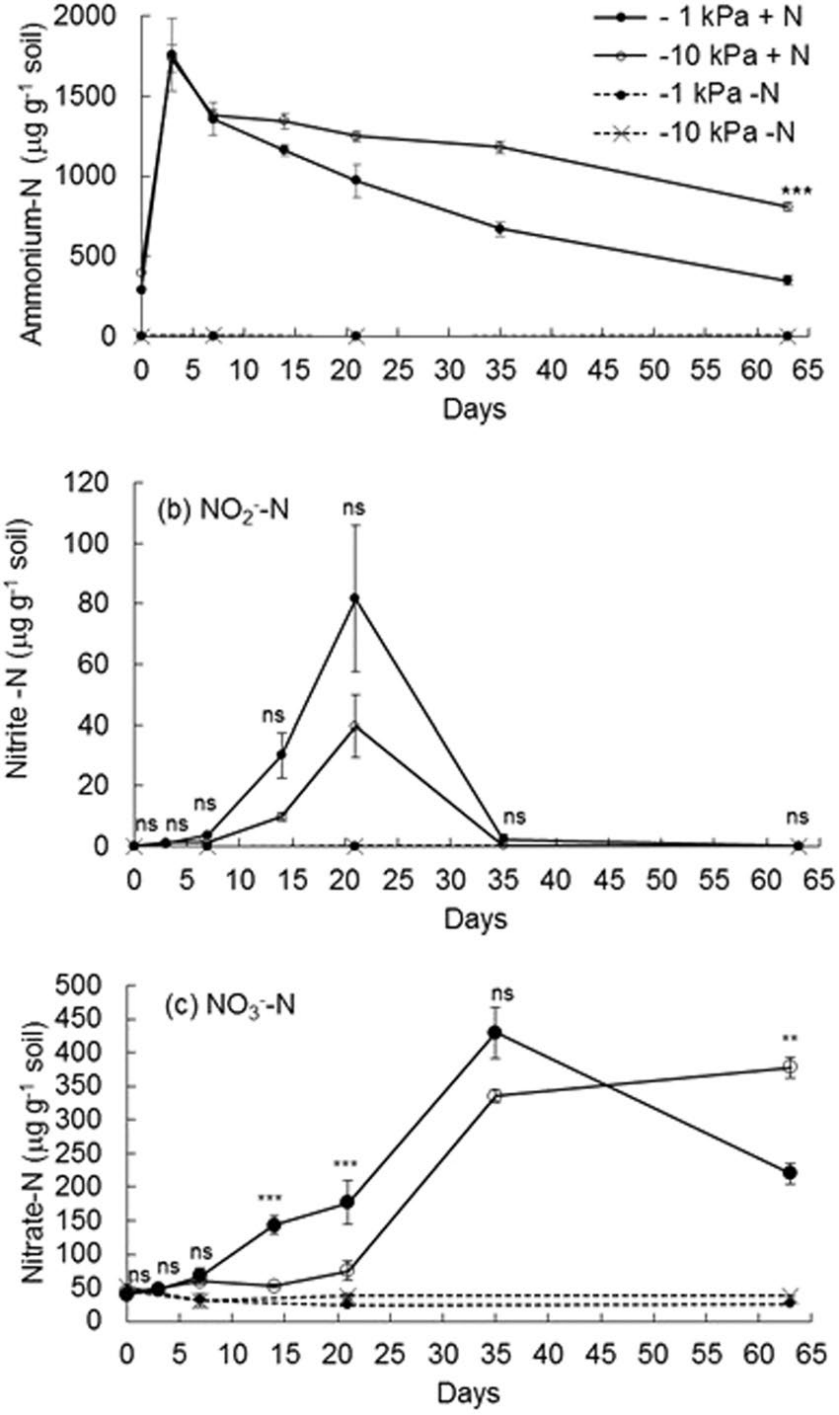
Changes in soil inorganic-N over time. Concentrations of extractable (**a**) ammonium-N (**b**) nitrite-N and (**c**) nitrate-N under near saturated (−1 kPa) or field capacity (−10 kPa) soil moisture conditions, following urea application (+N) or nil urea application (−N). Symbols are means (n = 4) with vertical error bars the standard error of the mean. Asterisks *^,^**^,^***indicate significant differences between moisture treatments under urea treatments at P < 0.05, P < 0.01, and P < 0.001, respectively.

The −1 kPa and −10 kPa moisture treatments imposed resulted in average WFPS values (%±s.e.m) of 88.9 ± 1.1 and 48.5 ± 0.4, respectively. The relative gas diffusivity values at −1 and −10 kPa were 0.0028 and 0.2079, respectively. There was a significant interaction of soil moisture and sampling date (p < 0.001) on soil pH, DOC and inorganic N contents (Figs 2–4). Soil pH in the non-urea treatment was generally constant over time (Fig. 2) regardless of soil moisture treatment, averaging 5.49 ± 0.11 (Stdev). However, soil pH (p < 0.001) increased within 6 hours of urea application, and increased further, peaking at 8.57 ± 0.29 and 8.78 ± 0.09 in the −1 kPa and −10 kPa treatments, respectively, on day 3 before declining over time (Fig. 2). On days 21 and 35 the soil pH was lower in the −1 kPa treatment than in the −10 kPa treatment (p < 0.001) with the reverse occurring on day 63 (p < 0.05).

Soil DOC was higher (P < 0.001) under the urea treatment throughout the experiment (Fig. 3) and within the urea treatment soil DOC concentrations were significantly lower at −1 kPa than at −10 kPa from day 3 to day 62 (Fig. 3). In the urea treatment soil DOC correlated strongly with soil pH at both −1 kPa (*r* = 0.79; p < 0.001) and −10 kPa (*r* = 0.89; p < 0.001).

**Figure 5:**
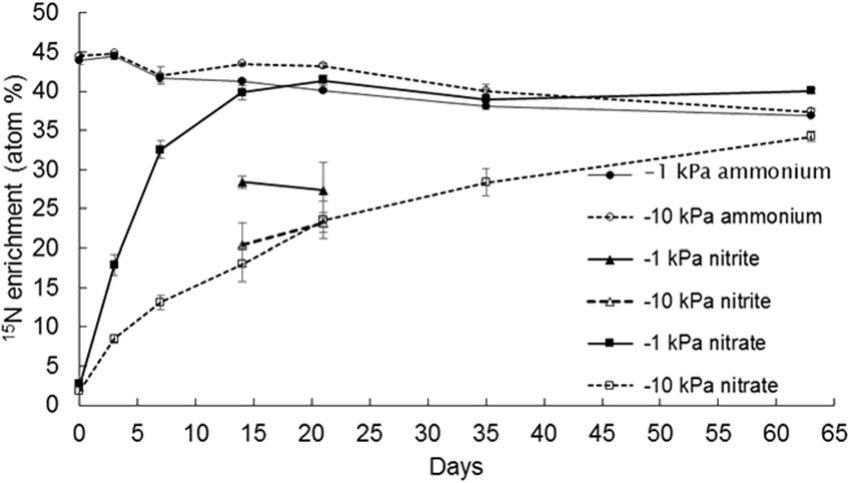
Inorganic-N ^15^N enrichment over time. The ^15^N enrichment of the ammonium-N (NH_4_^+^-N), nitrite-N (NO_2_^−^-N) and nitrate-N (NO_3_^−^-N) are shown over time following ^15^N urea application at near saturated (−1 kPa) or field capacity (−10 kPa) soil moisture conditions. Symbols are means (n = 4) with vertical error bars the standard error of the mean.

Soil NH_4_^+^-N concentrations increased following urea application (Fig. 4), peaking at day 3 and then declining over time with a faster rate of decline in the −1 kPa treatment from day 14 (p < 0.05) such that soil NH_4_^+^-N concentrations were lower at −1 kPa on days 35 and 63 (Fig. 4). The ^15^N enrichment of the NH_4_^+^-N in the urea treatment declined from 44 to 37 atom% over the experiment with higher ^15^N enrichment on days 14, 21 and 35 in the −10 kPa treatment (Fig. 5). Concentrations of NO_2_^−^-N increased from day 7 under the urea treatment and peaked at day 21, with more NO_2_^−^-N present in the −1 kPa treatment, prior to returning to background levels at day 35 (Fig. 4). Concentrations of NO_2_^−^-N, extracted from the urea treatment, were only sufficient for ^15^N enrichment determinations on days 14 and 21, where the ^15^N enrichment was higher (p < 0.05) at −1 kPa than at −10 kPa on day 14, with no differences on day 21 (Fig. 5). Soil NO_3_^−^-N concentrations also began to increase at day 7 under the urea treatment and were consistently higher (p < 0.001) in the −1 kPa treatment on days 14 and 21. Soil NO_3_^−^-N concentrations peaked on day 35, before they declined to be less than those observed in the −10 kPa treatment (p < 0.01) at day 63 (Fig. 4). Changes in soil NO_3_^−^-^15^N enrichment reflected the concentration dynamics with ^15^N enrichment increasing faster at −1 kPa to 41 atom% ^15^N at day 21 while at −10 kPa the NO_3_^−^-^15^N enrichment was only 34 atom% ^15^N by day 63 (Fig. 5).

### N_2_O-N fluxes and ^15^N enrichment

**Figure 6:**
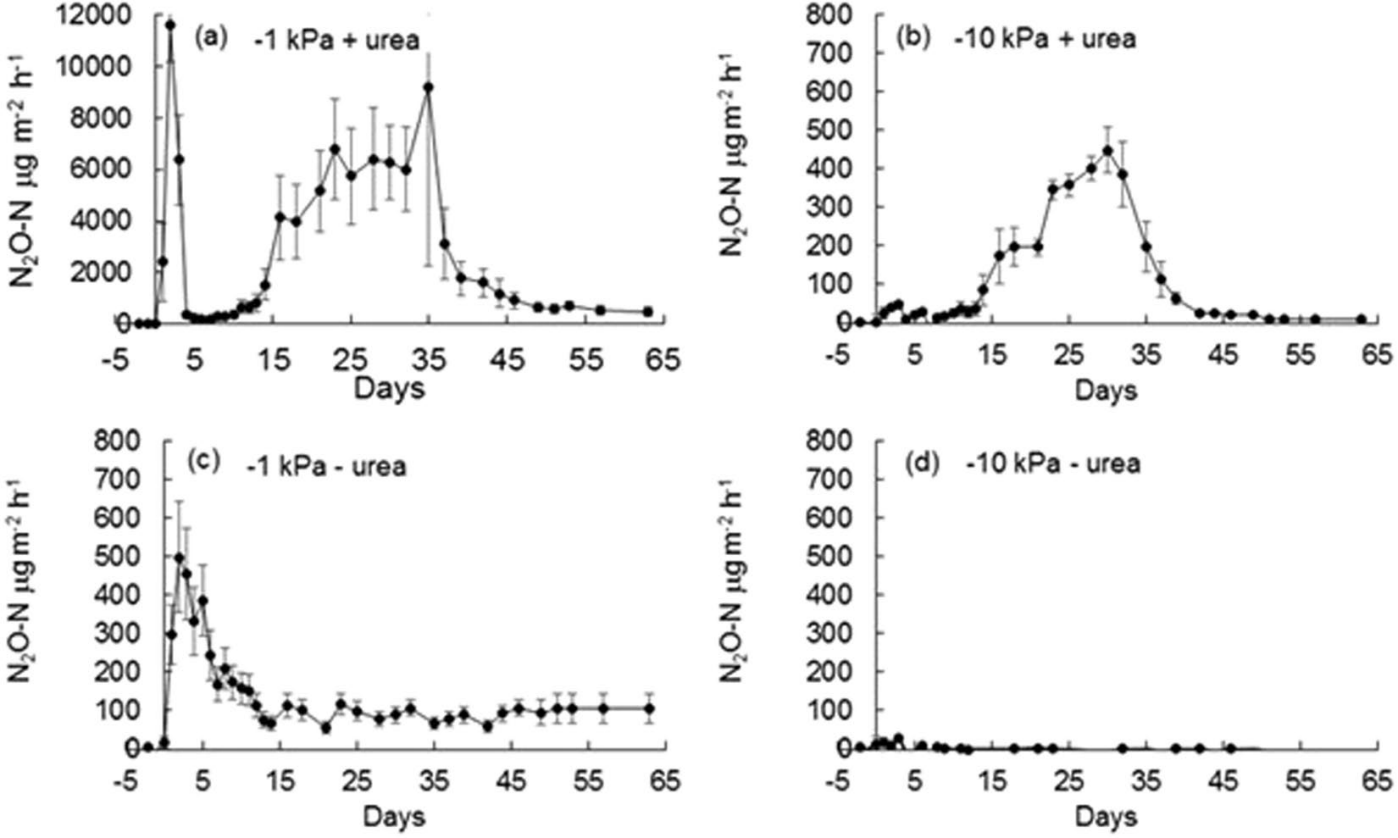
Nitrous oxide fluxes over time. Fluxes of N_2_O under near saturated (−1 kPa) or field capacity (−10 kPa) soil moisture conditions, following urea application (+N) or nil urea application (−N) where (**a**) −1 kPa +N (**b**) −10 kPa +N (**c**) −1 kPa –N and (**d**) −10 kPa –N show N_2_O fluxes over time. Note differing y-axis scales. Symbols are means (n = 4) with vertical error bars the standard error of the mean.

Trends in daily N_2_O fluxes differed with treatment (Fig. 6). At −10 kPa in the absence of urea N_2_O-N fluxes were generally <5 μg m^−2^ h^−1^, with fluxes only greater than this (≤29 μg m^−2^ h^−1^) between day 0 and day 10 following treatment application (Fig. 6). Under the −1 kPa treatment, in the absence of urea, N_2_O-N fluxes also peaked after water application on day 2 at 498 μg m^−2^ h^−1^, before declining to ca 100 μg m^−2^ h^−1^ on day 12, where after N_2_O-N fluxes were constant until day 63, averaging 92 μg N_2_O-N m^−2^ h^−1^ between days 12 to 63 (Fig. 6). Adding urea at −10 kPa caused N_2_O-N fluxes to increase steadily from day 12 until they peaked at day 30 (449 μg m^−2^ h^−1^) where after they steadily declined to <10 μg m^−2^ h^−1^ by day 51 (Fig. 6). The highest N_2_O-N fluxes were observed at −1 kPa with urea addition, where a rapid increase in the flux occurred peaking at 11,603 μg m^−2^ h^−1^ on day 2, followed by a rapid decrease to 163 μg m^−2^ h^−1^ by day 7. Then the flux gradually increased until day 35 (9220 μg m^−2^ h^−1^) whereupon it too decreased to be 476 μg m^−2^ h^−1^by day 61 (Fig. 6).

Soil moisture treatment influenced cumulative N_2_O-N fluxes (p < 0.001) with total emissions of 0.08 and 2.26 g N_2_O-N m^−2^ at −10 and −1 kPa, respectively, when averaged over plus and minus urea treatments. Similarly, application of urea increased cumulative N_2_O-N fluxes (p < 0.001) from 0.10 to 2.25 g N_2_O-N m^−2^ when averaged over soil moisture treatments. An interaction between soil moisture and N application (p < 0.002) resulted in higher cumulative N_2_O-N fluxes at −1 kPa when urea was applied equal to 3.99 g m^−2^ (Table 1). The N_2_O-N emission factors for the urea-N applied, allowing for non-N fluxes equated to 4.14% and 0.18% of N applied at −1 kPa and −10 kPa, respectively.

**Table 1:** Mean cumulative N_2_O, N_2DN_ and N_2_co emissions (g N m^−2^). P values are for the interaction between treatments.

Urea-N	Moisture (kPa)	N_2_O		N_2DN_		N_2_co	
+N	−1	3.99	A	8.61	A	1.92	A
+N	−10	0.18	B	1.98	A	0.26	B
−N	−1	0.16	B	na		na	
−N	−10	−0.0003	C	na		na	
P value		0.0321		0.0554		0.0437	

Tukey-Kramer grouping: LS-means with the same letter are not significantly different, na not applicable. N_2DN_ and N_2_co represent heterotrophic denitrification and codenitrification, respectively.

**Figure 7:**
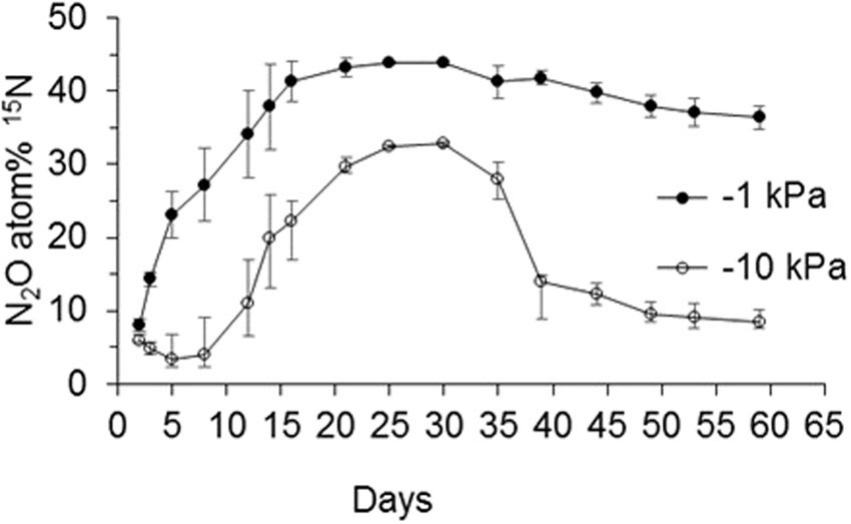
Nitrous oxide ^15^N enrichment over time. The ^15^N enrichment of the N_2_O molecule, over time, is shown for N_2_O evolved from soil under near saturated (−1 kPa) or field capacity (−10 kPa) conditions, following ^15^N urea application. Symbols are means (n = 4) with vertical error bars the standard error of the mean.

**Figure 8:**
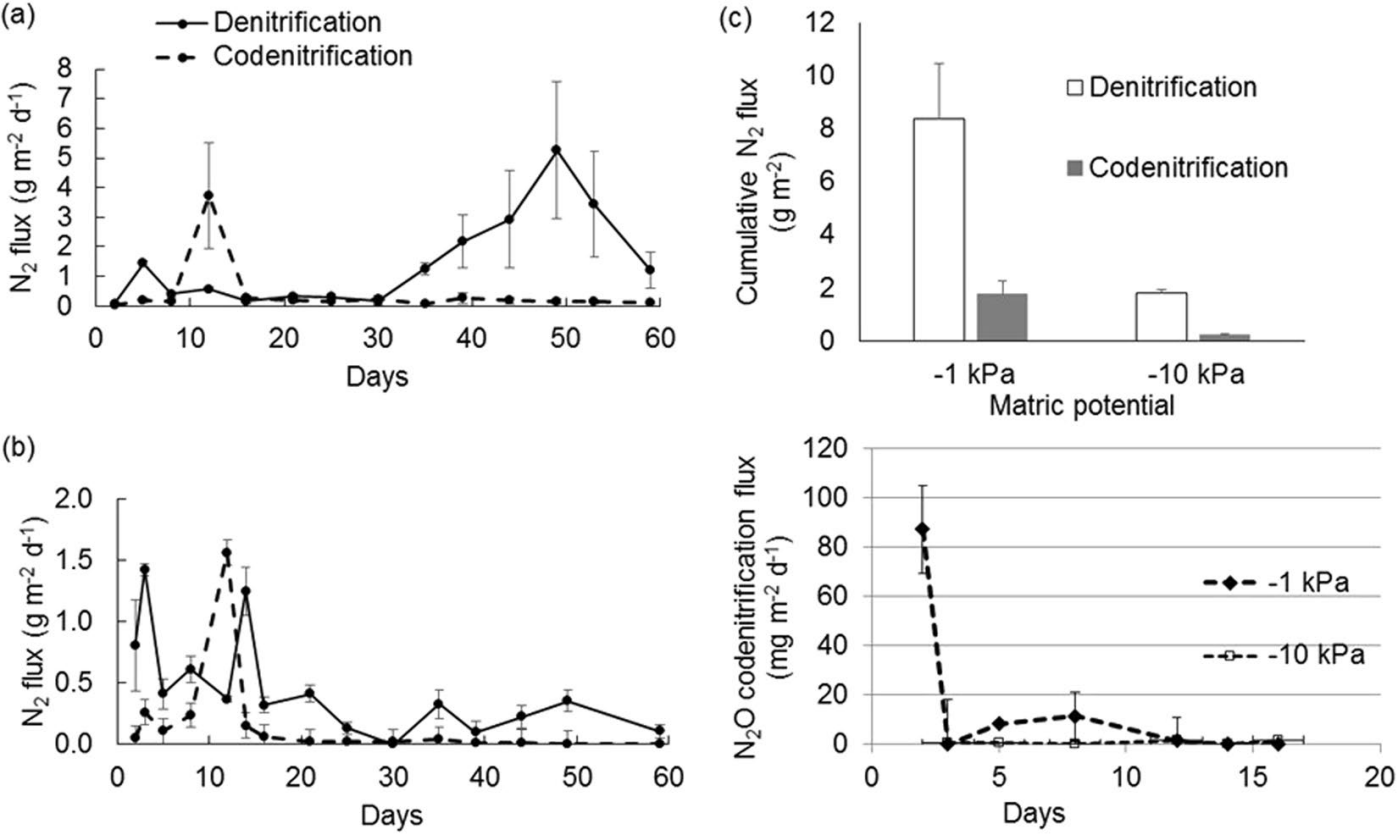
Denitrification and codenitrification fluxes over time. The codenitrification and denitrification fluxes, over time since ^15^N urea addition, are shown as daily N_2_ fluxes for (**a**) soil at −1 kPa (**b**) soil at −10 kPa and (**c**) as cumulative codenitrification and denitrification N_2_ fluxes, while (**d**) is the N_2_O codenitrification flux, over time since ^15^N urea addition, as daily N_2_O fluxes. Symbols are means (n = 4) with vertical error bars the standard error of the mean.

Upon urea application, the atom % ^15^N enrichment of the N_2_O emitted at −1 kPa increased steadily to reach a maximum value of 43.9 atom % ^15^N on day 25 before declining at a relatively slow rate to a value of 36.3 atom % ^15^N by day 59 (Fig. 7). With the exception of day 2, the atom % ^15^N enrichment of the N_2_O emitted at −1 kPa was higher than that emitted at −10 kPa (P < 0.05) on any given day. At −10 kPa the atom % ^15^N enrichment of the N_2_O flux was observed to increase abruptly at day 12, reaching a maximum of 32.8 on day 30 and thereafter declining relatively abruptly to remain at ca 10 atom % ^15^N (Fig. 7). Fluxes of N_2_O associated with codenitrification were low and only measurable on days 2, 5, 8 and 12 for the −1 kPa treatment and days 3, 5, 8, 12 and 16 for the −10 kPa treatment (Fig. 8). Highest fluxes were observed for the −1 kPa treatment (3637 μg N_2_O-N m^−2^ hr^−1^) comprising 20% of total N_2_O flux with emissions of codenitrified N_2_O subsequently reducing rapidly. Codenitrified N_2_O fluxes in the −10 kPa treatment were extremely low and never rose above 70 μg N_2_O-N m^−2^ hr^−1^).

### N_2_ fluxes and codenitrification after urea addition

The average daily denitrification fluxes were 1.48 (0.34) g N m^−2^ d^−1^ and 0.53 (0.07) g m^−2^ d^−1^ (s.e.m in brackets) at −1 and −10 kPa, respectively. At −1 kPa denitrification fluxes initially peaked at day 5 and then were higher after day 30, peaking on day 49 (Fig. 8). The decline in soil NO_3_^−^ concentration after day 35 coincided with higher denitrification fluxes. At −10 kPa denitrification fluxes were highest after the initial wetting up following treatment application where after they generally declined (Fig. 8). Consequently, cumulative denitrification as N_2_ was higher (p = 0.055) at −1 kPa, totaling 8.61 g N m^−2^, than at −10 kPa where observed fluxes were 1.98 g N m^−2^ (Fig. 8).

The average daily codenitrification fluxes under urea treatments at −1 and −10 kPa were 0.38 (0.15) g N m^−2^ d^−1^ and −10 kPa 0.07 (0.01) g N m^−2^ d^−1^, respectively. Codenitrification fluxes peaked on day 12 regardless of soil kPa value, but were higher on day 10 at −1 kPa (Fig. 8). Average daily codenitrification fluxes were ca 5-fold higher at −1 kPa after day 30 than at −10 kPa. Consequently, cumulative codenitrification rates of N_2_ were also higher (p = 0.043) at −1 kPa (1.91 g N m^−2^) than at −10 kPa (0.26 g N m^−2^). Cumulative codenitrification, as a proportion of denitrification, did not vary as a result of soil matric potential equaling 25.5 ± 15.8% and 12.9 ± 4.8% (±stdev) at −1 and −10 kPa, respectively. The contribution of codenitrification as a proportion of total denitrification (codenitrification plus denitrification) also did not vary with soil matric potential, being 19.3 ± 10.4% and 11.3 ± 3.8% (±stdev) at −1 and −10 kPa, respectively.

## Discussion

### Inorganic-N pools and ^15^N enrichment

Following urea application to the soil the ensuing hydrolysis produces NH_4_^+^ and bicarbonate (HCO_3_^−^) ions. The HCO_3_^−^ ions are further hydrolysed to produce hydroxide ions (OH^−^) and carbon dioxide^25^ and it is this second hydrolysis reaction that generated the observed increase in soil pH under the urea treatments (Fig. 2). Elevated soil pH also influences the equilibrium between NH_4_^+^ and ammonia (NH_3_): as soil pH becomes elevated (>7.0) concentrations of NH_3_ increase^25^. Urea-N not volatilized as NH_3_ may be transferred along the inorganic-N cascade via NH_4_^+^, NO_2_^−^ and NO_3_^−^.

During nitrification microbes utilise NH_4_^+^ and oxidise it to NO_2_^−^. Elevated NH_3_ concentrations may inhibit NH_4_^+^ oxidation^26,27^. Thus the slower decline in the NH_4_^+^ concentration observed in the −10 kPa treatment, under urea, may have been due to NH_3_ inhibition of nitrification. In favour of this were both the relative gas diffusivity of the soil being 2 orders of magnitude higher at −10 kPa, which would have facilitated NH_3_ diffusion through the soil, and the soil pH remaining higher for longer (Fig. 2). The latter would have promoted the presence of NH_3_ for longer. A slower rate of decline in soil pH at −10 kPa also demonstrates nitrification was slower, since nitrification results in the net release of H^+^ ions^19^. Further evidence to support a slower rate of NH_4_^+^ oxidation can be found in the slower rate of increase in ammonium oxidizing bacteria (*AOB*) gene and transcript abundance^28^.

Elevated soil NO_2_^−^ concentrations resulted from nitrification of NH_4_^+^ and their increase, from day 5 until day 20, occurred over a period when soil pH was sufficiently high to result in NH_3_ generation. Ammonia toxicity acts more strongly on nitrifier NO_2_^−^ oxidation than nitrifier NH_4_^+^ oxidation^29^. It has been shown that solution-phase NH_3_ (*sl*NH_3_) inhibits NO_2_^−^ oxidation, as evidenced by strong relationships between cumulative *sl*NH_3_ and cumulative NO_2_^−^ and static copy numbers of the *nxr*A gene, which is associated with nitrite oxidoreductase, and as a consequence soil NO_2_^−^ is strongly correlated with N_2_O production^29^. The high N_2_O fluxes that occurred, between ca. days 7 to 35, at both −1 and −10 kPa under urea, where the soil NO_2_^−^ concentrations were elevated strongly demonstrates this, and it can be assumed *sl*NH_3_ induced NO_2_^−^ toxicity lead to the ensuing N_2_O emissions.

The higher NO_3_^−^ concentrations observed under urea on days 14 and 21 at −1 kPa were a consequence of the more rapid nitrification rates in this treatment, while the lower NO_3_^−^ concentration in this treatment observed at day 63 resulted from higher denitrification induced losses of NO_3_^−^, which is further supported by the increase in soil pH under this treatment, since denitrification results in a net release of OH^−^ ions^19^.

The ^15^N enrichment of the NH_4_^+^ pool, under urea, shows that it was predominantly derived from the urea applied, regardless of soil moisture treatment. The fact the NH_4_^+^ pool ^15^N enrichment was initially ca. 5 atom% lower than the urea solution applied was likely due to the release of NH_4_^+^ as a consequence of the high soil pH solubilising soil organic matter, as demonstrated by the elevated DOC concentrations under the urea treatment. Solubilisation of soil organic matter is routinely observed following urine or urea application to soil^30^. The reason for the NO_2_^−^ pool ^15^N enrichment being ca. half that observed in the NH_4_^+^ pool on days 14 and 21 at −1 kPa, shows antecedent soil N was also contributing to this pool which could have come from mineralization and subsequent oxidation of NH_4_^+^, despite the presence of NH_3_, since relatively low quantities of NH_4_^+^ would be needed to dilute the NO_2_^−^ pool, or alternatively there may have been some denitrification of antecedent NO_3_^−^ generating NO_2_^−^. The fact that the NO_3_^−^ pool ^15^N enrichment aligned closely with that of the NO_2_^−^ pool ^15^N enrichment at −10 kPa demonstrates NO_2_^−^ was the dominant precursor to NO_3_^−^ pool at −10 kPa. Furthermore, the slower rate of increase in the NO_3_^−^ pool ^15^N enrichment at −10 kPa, when compared to −1 kPa, further supports the fact there was a slower rate of nitrification at −10 kPa. The increase in the NO_3_^−^ pool ^15^N enrichment over time, in both the −1 and −10 kPa treatments, demonstrates the NO_3_^−^ pool was initially dominated by antecedent soil NO_3_^−^ as in fact occurred (Fig. 4c).

### N_2_O fluxes and ^15^N enrichment

While simply wetting of the soil, as occurred under the non-urea treatment, induced N_2_O fluxes at −1 kPa, this wetting effect was not sufficient to generate the high N_2_O fluxes observed under urea from days 0 to 4. These high initial N_2_O fluxes under urea, as previously observed^31^, are due to the chemically induced anoxia that results from the hydrolysis reactions generating both NH_3_ and CO_2_, as demonstrated *in situ*^32^. Such high fluxes were not observed at −10 kPa during this period because the higher relative gas diffusivity of the soil at −10 kPa ensured the soil was not as anaerobic.

As noted above periods of high N_2_O flux between days 14 and 37 aligned with the presence of elevated NO_2_^−^ concentrations. The atom % ^15^N enrichment of the N_2_O at −10 kPa was comparable with that of the NO_2_^−^ pool at this time, further demonstrating that the N_2_O flux predominately originated from the NO_2_^−^ pool, and because the ^15^N enrichment of the N_2_O declined as NO_2_^−^ concentrations declined. Despite both the NO_3_^−^ concentration and NO_3_^− 15^N enrichment both increasing after this time, this was not reflected in any increased N_2_O fluxes or its ^15^N enrichment because the higher relative gas diffusivity at −10 kPa made conditions unsuitable for the denitrification of NO_3_^−^ ^24^.

However, at −1 kPa the N_2_O evolved predominately via denitrification of the NO_3_^−^ pool up until ca. day 15 as demonstrated by the alignment of the N_2_O ^15^N enrichment with the NO_3_^−^ pool ^15^N values. The higher N_2_O fluxes at −1 kPa between days 15 to 35 were ca. 15-fold higher due to the more anaerobic conditions and, as inferred above, are presumed to have occurred as a result of the relatively high NO_2_^−^ concentrations over this period. However, the N_2_O ^15^N enrichment did not reflect that of the KCl extracted NO_2_^−^ pool measured on days 14 and 21 at −1 kPa, but did reflect that of the NH_4_^+^ and NO_3_^−^ pools on these days. Differences in the ^15^N enrichment of the KCl extracted NO_2_^−^ and actual *in situ* ^15^N enrichment of the NO_2_^−^ pool may possibly have arisen due to the method of treatment application where, in the −10 kPa treatment the urea solution infiltrated further and contacted a greater soil volume than at −1 kPa, as evidenced by the greater release of DOC at −10 kPa (Fig. 3), and which would have resulted in a more uniform NO_2_^−^ pool. It is likely that, at −1 kPa, denitrification of antecedent NO_3_^−^ occurred and that this generated sufficient NO_2_^−^ to isotopically dilute the relatively small ^15^N enriched NO_2_^−^ pool, derived from NH_4_^+^ and/or NO_3_^−^, when the soil was extracted. After day 35, the N_2_O ^15^N enrichment reflected that of the NO_3_^−^ pool, and given the compatible conditions for denitrification, it can be assumed that denitrification of the NO_3_^−^ pool dominated N_2_O production after day 30, and this assumption is supported by the elevated denitrification flux occurring after this time (Fig. 8).

### N_2_ denitrification and codenitrification of N_2_ and N_2_O

As expected denitrification occurred at higher rates under the more anaerobic moisture treatment as a result of the lower *Dp/Do* conditions promoting denitrification in the presence of NO_3_^−^ substrate.

The N transformations that ensued following urea hydrolysis, and hydrolysis itself, generated previously recognized codenitrification nucleophiles that include NH_4_^+^, NH_3_, and possibly organic-N compounds such as amines^16^. The latter might occur as a result of the dissolution of soil organic matter. While the enzymatically utilized NO_2_^−^ and NO compounds, that form electrophiles, are generated during nitrification and denitrification^19^.

Codenitrification N_2_O fluxes were generally low for both treatments, with measurable values mainly associated with the initial soil wetting. Conversely, codenitrification to N_2_ was observed to peak on day 12, regardless of soil moisture, when NH_3_, NH_4_^+^ and NO_2_^−^ were all present at an elevated soil pH (≥7.70), and at relatively high concentrations. Thus it is possible that either NH_3_ or NH_4_^+^ were undertaking the role of the nucleophile at this time, since the elevated pH (>5.5) would have prevented any significant abiotic nitrosation occurring via NO^+^ formation^16^. Recently, however, the formation of both N_2_O and N_2_, under both oxic and anoxic conditions, was reported in an *in vitro* experiment maintained at pH 6.2–6.9 where either live fungi or fungal necromass were incubated with glutamine and NO_2_^−^ ^33^. A subsequent isotope experiment with glutamine and ^15^NO_2_^−^ demonstrated the hybrid formation of N_2_ after an incubation period of >7 days, again under either oxic or anoxic conditions^33^. Hence, based on this recent study, even though the soil in the current study was at a pH (≥7.70) sufficient to prevent acidic pathways of abiotic hybrid N-N bonds forming, we cannot rule out the possibility that abiotic reactions, under alkaline conditions, contributed to the codenitrification flux measured in the current experiment.

Production of N_2_O or N_2_ via biotic codenitrification may result from the actions of archaea, bacteria or fungi. While archaea have been found to generate N_2_O through N-nitrosating hybrid formation^34^ they are unlikely to have been the dominant mechanism in the current study since archaea are thought to prefer low N conditions^35,36^ and urea addition resulted in lower ammonia oxidizing archaea gene copy numbers^28^. The codenitrification observed is most likely to be the result of fungi or bacterial activity. Delineation of the relative contributions made by fungi or bacteria to codenitrification is beyond the scope of the present study, however, future studies should aim to examine relative fungal and bacterial contributions.

Spott *et al*.^16^ conceptualized that the recognized constraints on denitrification might also apply to codenitrification, and thus higher codenitrification fluxes might be expected under more anaerobic conditions. The current results support this concept: after day 30 the higher daily codenitrification fluxes under the more anaerobic (−1 kPa) soil moisture conditions, when at the same time denitrification fluxes were higher, resulted in higher cumulative codenitrification fluxes. This reinforces the fact that NO_2_^−^ and or NO play a key role in the codenitrification process. The NO molecule has been observed to readily diffuse within the soil profile^37^, at relatively high concentrations, during denitrification and this would result in reactions with nucleophiles.

Unlike the results of Selbie *et al*.^23^ codenitrification did not dominate the N_2_ fluxes observed in the current study. This could be the result of the experimental system used in the current study differing to that used by Selbie *et al*.^23^. Differences include the lack of a pasture turf and associated microbiology and root exudation, the use of sieved repacked soil that may also have altered the fungal-bacterial community structure or activity as a result of sieving, constant soil moisture contents as opposed to wetting and drying events, and the lack of other climatic variables such as wind and rainfall.

In particular, fungal populations may have been reduced on sieving, and given that fungal P450 NOR is implicated in supplying enzyme bound nitrosating agents this could have had a significant influence on the results^38^. Given that enzyme bound nitrosating agents produced during denitrification may also consist of metal-nitrosyl complexes^16^ any differences in soil Fe and Cu levels between studies may also explain the observed differences in codenitrification. Likewise, differences in the kinetic properties of different nucleophiles, combined with the ratio of NO or NO_2_^−^ availability to nucleophile concentration, have also been shown to significantly impact on codenitrification/denitrification: lower *K*_*m*_ and high nitrosyl donor/nucleophile ratios have been shown to reduce the level of codenitrification^15,20^.

This study confirms the role of anaerobic soil conditions in enhancing codenitrification fluxes under ruminant urine/urea deposition. It also demonstrates for the first time that high levels of NO_2_^−^, or other transitional N compounds ensuing from NO_2_^−^, that may occur during nitrification, are also able to contribute to codenitrification processes. To progress knowledge of codenitrification in grazed pastures more detailed studies are now required to both identify the microbial pathways operating and the relative importance of the possible nucleophiles and nitrosating agents that occur in grazed pastures.

## Materials and Methods

### Soil collection and experimental design

Soil was collected in early spring (March) from a permanently grazed dairy pasture at the Teagasc Moorepark Research Centre, County Cork, Ireland (8°15′W, 52°9′N). The top 5 cm of soil was removed and the A-horizon was sampled, 5–20 cm depth. Soil physical and textural characteristics are shown in Table 2. Cows had not grazed the pasture for over one month so recent urine deposition sites were avoided. The soil is classified as a Typical Brown earth from the Clashmore Series^39^, or as a Haplic Cambisol in the World Reference Database^40^. Field moist soil was then bagged and shipped to Lincoln University, New Zealand, following appropriate biosecurity protocols. It was then sieved (≤2 mm) to remove any stones, plant roots or earthworms. Sieved soil, with a gravimetric water content (*θg*) of 0.24 g water g^−1^ soil, was then packed into stainless steel rings (7.3 cm internal diameter, 7.4 cm deep) to a depth of 4.1 cm at a bulk density of 1.1 Mg m^−3^, the latter simulating the *in situ* soil bulk density. This resulted in a total porosity of 0.58 cm^3^ pores cm^−3^ soil. Packed soil cores were then arranged in a factorial experiment replicated four times.

**Table 2:** Physical and textural characteristics of soil sampled.

Depth	Bulk density (Mg m^−3^)	Porosity (%)	Texture	Sand (%)	Silt (%)	Clay (%)
0–10	1.19	0.55	Sandy loam	53	31	16
10–20	1.28	0.52	Sandy loam	55	31	14

Treatments consisted of two levels of soil moisture, −1 kPa and −10 kPa simulating ‘near-saturation’ and ‘field-capacity’, respectively, and two levels of urea, (0 and 1000 kg N ha^−1^), replicated 4 times, with 7 destructive sampling times (112 cores in total). Preliminary tests showed that −1 and −10 kPa corresponded to 53% and 30% volumetric water content, or 91% and 52% water-filled pore space (WFPS). Soil cores were maintained at these water contents using tension tables^41^. Soil relative gas diffusivity values were calculated using the values for air-filled pore space and total porosity and the generalized-density corrected equation of Chamindu Deepagoda *et al*.^42^; Equation 9b. It is recognized that artificial urine simulation does not generate identical effects to ruminant urine^43^, that urea contributes >70% of the total urine-N pool^6,44^, and that this N source is predominately responsible for the subsequent dynamics and transformations of organic and inorganic N in the soil under ruminant urine patches. Thus, in order to apply the N treatments, soil cores were wetted up on the tension tables to a point where there remained the capacity to add a further 10 mL of liquid, without inducing drainage. Subsequently, in the plus N treatment, 10 mL of a urea solution (42 g urea-N L^−1^; 50 atom%, Cambridge Isotope Laboratories Inc., USA) was slowly applied to the soil surface, to avoid drainage, to mimic an extreme bovine urine deposition event with a potentially high N_2_ flux. Real urine could not be used since there was a need to have the urea-N highly enriched with ^15^N to detect N_2_ fluxes. In the nil N treatment 10 mL of deionized water was applied instead of a urea solution. Tension tables were maintained in a room with a mean temperature of 20 °C.

### Soil chemical analyses

After treatment application and throughout the experiment, on days 0, 3, 7, 14, 21, 35, and 63, soil inorganic N concentrations were determined by destructively sampling 16 soil cores (2 levels of urea × two levels of soil moisture × 4 replicates). Soil cores were fully extracted, homogenized, and a subsample was taken to determine *θg*: by drying the soil at 105 °C for 24 hours. A flat surface pH electrode was used to determine soil pH (Broadley James Corp., Irvine, California). Then further soil subsamples were extracted (equivalent of 10 g dry soil: 100 mL 2 M KCl shaken for 1 hour) and filtered (Whatman 42) to determine soil inorganic-N. The NH_4_^+^-N, NO_2_^−^-N, and NO_3_^−^-N concentrations were analysed using flow injection analysis^45^. The ^15^N enrichment of NH_4_^+^-N was determined according to Stark and Hart^46^ while NO_2_^−^-^15^N and NO_3_^−^-^15^N enrichments were determined according to the methods of Stevens and Laughlin^47^. Concentrations of dissolved organic carbon (DOC) in the soil were measured according to Ghani *et al*.^48^ with analyses performed on a Shimadzu TOC analyzer (Shimadzu Oceania Ltd., Sydney, Australia).

### Gas flux determinations

Nitrous oxide and N_2_ fluxes were regularly determined, from two days before until 63 days after treatment application using only the last batch of soil cores to be destructively analysed. This was performed by placing a soil core into a 1-L stainless steel tin fitted with a gas-tight lid and rubber septa. Samples for N_2_O flux determinations were taken upon lid closure and then after 15 and 30 minutes. A further sample was taken for N_2_O-^15^N enrichment and N_2_ flux determination after 3 hours, after which cores were returned to the tension tables. Gas samples were taken using a 20-mL glass syringe fitted with a 3-way tap and a 0.5 mm by 16 mm needle and placed in either 6 mL vials for the N_2_O flux determinations or 12 mL vials for the N_2_O-^15^N enrichment and N_2_ flux samples (Exetainer; Labco Ltd., Lampeter, UK). An automated gas chromatograph (8610; SRI Instruments, Torrance, CA), coupled to an autosampler (Gilson 222XL; Gilson, Middleton, WI), was used to determine N_2_O gas concentrations in the samples, as previously described^49^. A continuous-flow-isotope mass spectrometer (Sercon 20/20; Sercon, Chesire, UK) inter-faced with a TGII cryofocusing unit (Sercon, Chesire, UK), was used to determine the ^15^N enrichment of the N_2_O-N and N_2_-N gas samples^50^.

The ion currents (I) at mass to charge ratios (m/z) of 44, 45, and 46 facilitated the calculation of the N_2_O molecular mass ratios ^45^R (^45^I/^44^I) and ^46^R (^46^I/^44^I). The N_2_O sources were subsequently allocated to either the fraction derived from the denitrifying pool (*d’*_*D*_) of enrichment *aD* or the fraction derived from the pool or pools at natural abundance d’_N_ = (1-*d’*_*D*_) using the method of Arah (1997). The ion currents at m/z 28, 29, and 30 permitted the N_2_ molecular ratios ^29^R (^29^I/^28^I) and ^30^R (^30^I/^28^I) to be quantified. Differences between the N_2_ molecular ratios of the enriched and ambient atmospheres were expressed as Δ^29^R and Δ^30^R The N_2_ flux was subsequently calculated using three methods:

(i) The enrichment of the denitrifying pool (^15^X_N_) was calculated using Δ^29^R and Δ^30^R, and then the N_2_ flux^51^,
(ii) Using only the Δ^30^R data with the assumption that the enrichment of the denitrifying pool was *aD*^52^ and the equation of Mulvaney^53^
(iii) Using Δ^29^R and Δ^30^R to calculate the relative contributions of denitrification (N_2DN_), according to method (ii), and codenitrification (N_2CO_).

Increases in Δ^29^R and Δ^30^R may occur from denitrification but codenitrification contributes most to Δ^29^R where the ratio of Δ^29^R to Δ^30^R is always 272^54^. By assuming all Δ^30^R was the result of denitrification, method (ii), N_2DN_ was calculated. Then using the ‘backsolver’ facility in Microsoft Excel^TM^, the contribution of Δ^29^R to N_2DN_ was determined. The difference between the total measured value of Δ^29^R and Δ^29^R determined for N_2DN_ was assigned to codenitrification. Thus the fraction of the total number of moles of N_2_ in the headspace, resulting from codenitrification (*d*_*CD*_) were calculated as:

1 $${d}_{CD}=-{{\rm{\Delta }}}^{29}R{p}_{1}^{2}/(-{{\rm{\Delta }}}^{29}R{p}_{1}^{2}+{{\rm{\Delta }}}^{29}R{p}_{1}{p}_{2}+{q}_{1}{p}_{2}-{q}_{2}{p}_{1})$$

where *p*_*1*_ (0.9963) and *q*_*1*_ (0.0037) represent the atom fractions of ^14^N and ^15^N in the natural abundance pool, respectively, and *p*_*2*_ and *q*_*2*_ are the atom fractions of ^14^N and ^15^N in the enriched NO_3_^−^ pool, respectively, from which codenitrification is assumed to occur. Using the headspace volume of the sample chamber, corrected for standard temperature and pressure, the mass of N_2_-N in the headspace was determined with the amount derived from denitrification or codenitrification ascertained by multiplying by *d*_D_ or *d*_*CD*_, respectively.

### Data analyses

Data were analysed using the Glimmix procedure within the SAS® software version 9.4 (SAS, 2014). Cumulative results were analysed for the +N treatment only. For all other variables, analyses was as N treatment × moisture × day or moisture × day factorials. Any repeated measurements over time were modelled using correlation structures and spatial covariance was used to model the unequally-spaced time measurements. Residual checks were made and, where required, log transformation was used to correct for skew and non-constant variance. Multiplicity adjustments were made for simple effects within interactions, as interest was primarily in comparisons within time points.
