# Author Correction: Influence of soil moisture on codenitrification fluxes from a urea-affected pasture soil

**DOI:** 10.1038/s41598-018-22645-7

**Published:** 2018-03-07

**Authors:** Timothy J. Clough, Gary J. Lanigan, Cecile A. M. de Klein, Md. Sainur Samad, Sergio E. Morales, David Rex, Lars R. Bakken, Charlotte Johns, Leo M. Condron, Jim Grant, Karl G. Richards

**Affiliations:** 1 Department of Soil and Physical Sciences, Lincoln University, Lincoln, New Zealand; 2 Teagasc, Environmental Research Centre, Johnstown Castle, Wexford, Ireland; 3 AgResearch Invermay, Mosgiel, New Zealand; 4 Department of Microbiology and Immunology, Otago School of Medical Sciences, University of Otago, Dunedin, New Zealand; 5 Department of Environmental Sciences, Norwegian University of Life Sciences, Ås, Norway; 6 Statistics and Applied Physics, Teagasc, Ashtown, Dublin 15, Ireland

Correction to: *Scientific Reports* 10.1038/s41598-017-02278-y, published online 19 May 2017

This Article contains an error in Figure 3, where the y-axis ‘DOC (μg g^-1^ soil)’ is incorrectly labelled as ‘DOC (mg g^−1^ soil)’. The correct Figure 3 appears below as Figure 1.

**Figure 1:**
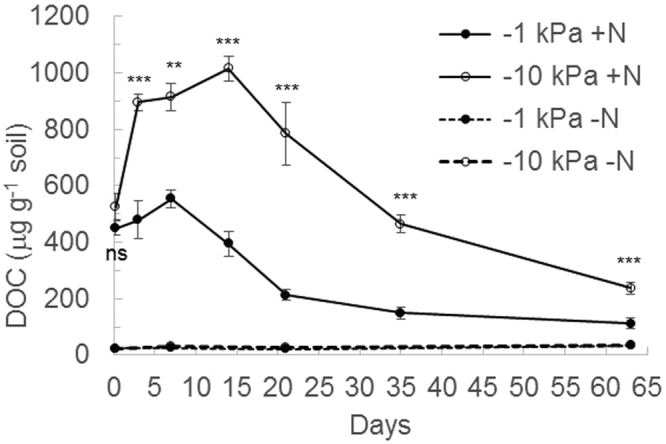
Changes in soil cold water extractable organic carbon (DOC) over time. Concentrations of soil DOC under near saturated (−1 kPa) or field capacity (−10 kPa) soil moisture conditions, following urea application (+N) or nil urea application (−N). Symbols are means (n = 4) with vertical error bars the standard error of the mean. Asterisks *’**’***indicate significant differences between moisture treatments under urea treatments at P < 0.05, P < 0.01, and P < 0.001, respectively.

